# ‘Barcode fishing’ for archival DNA from historical type material overcomes taxonomic hurdles, enabling the description of a new frog species

**DOI:** 10.1038/s41598-020-75431-9

**Published:** 2020-11-05

**Authors:** Mark D. Scherz, Safidy M. Rasolonjatovo, Jörn Köhler, Loïs Rancilhac, Andolalao Rakotoarison, Achille P. Raselimanana, Annemarie Ohler, Michaela Preick, Michael Hofreiter, Frank Glaw, Miguel Vences

**Affiliations:** 1 Zoologisches Institut, Technische Universität Braunschweig, Mendelssohnstr. 4, 38106 Braunschweig, Germany; 2 Zoologische Staatssammlung München (ZSM-SNSB), Münchhausenstr. 21, 81247 München, Germany; 3 Mention Zoologie et Biodiversité Animale, Université d’Antananarivo, BP 906, 101 Antananarivo, Madagascar; 4 Association Vahatra, Lot V A 38 LBA Ter Ambohidempona Tsiadana, BP 3972, 101 Antananarivo, Madagascar; 5 Hessisches Landesmuseum Darmstadt, Friedensplatz 1, 64283 Darmstadt, Germany; 6 School for International Training, VN 41A Bis Ankazolava Ambohitsoa, 101 Antananarivo, Madagascar; 7 Museum National d’Histoire Naturelle, ISYEB, CNRS, SU, EPHE, UA, 57 Rue Cuvier, 75005 Paris, France; 8 Institut für Biochemie und Biologie, Universität Potsdam, Karl-Liebknecht-Str. 24-25, 14476 Potsdam, Germany; 9 Present Address: Department of Evolutionary Biology, Universität Konstanz, Universitätsstr. 10, 78464 Konstanz, Germany

**Keywords:** Taxonomy, Zoology, Herpetology

## Abstract

Taxonomic progress is often hindered by intrinsic factors, such as morphologically cryptic species that require a broad suite of methods to distinguish, and extrinsic factors, such as uncertainties in the allocation of scientific names to species. These uncertainties can be due to a wide variety of factors, including old and poorly preserved type specimens (which contain only heavily degraded DNA or have lost important diagnostic characters), inappropriately chosen type specimens (e.g. juveniles without diagnostic characters) or poorly documented type specimens (with unprecise, incorrect, or missing locality data). Thanks to modern sequencing technologies it is now possible to overcome many such extrinsic factors by sequencing DNA from name-bearing type specimens of uncertain assignment and assigning these to known genetic lineages. Here, we apply this approach to frogs of the *Mantidactylus ambreensis* complex, which was recently shown to consist of two genetic lineages supported by concordant differentiation in mitochondrial and nuclear genes. These lineages co-occur on the Montagne dʼAmbre Massif in northern Madagascar but appear to have diverged in allopatry. We use a recently published bait set based on three mitochondrial markers from all known Malagasy frog lineages to capture DNA sequences from the 127-year-old holotype of *Mantidactylus ambreensis* Mocquard, 1895. With the obtained sequences we are able to assign the name *M. ambreensis* to the lowland lineage, which is rather widespread in the rainforests of northern Madagascar, leaving the microendemic high-elevation lineage on Montagne d’Ambre in north Madagascar in need of description. We describe this species as *Mantidactylus ambony* sp. nov., differing from *M. ambreensis* in call parameters and a smaller body size. Thus, using target enrichment to obtain DNA sequence data from this old specimen, we were able to resolve the extrinsic (nomenclatural) hindrances to taxonomic resolution of this complex. We discuss the broad-scale versatility of this ‘barcode fishing’ approach, which can draw on the enormous success of global DNA barcoding initiatives to quickly and efficiently assign type specimens to lineages.

## Introduction

A substantial, albeit inestimable, portion of the potentially over 100 million eukaryote species awaiting taxonomic description^1^ cannot be easily described due to some kind of intrinsic or extrinsic hindrance to taxonomy. Intrinsic hindrances, which we define as properties of the species themselves that complicate taxonomy, such as morphologically cryptic species, can be overcome by diversification of data types in species delimitation; in most cases, diagnostic features of cryptic species can be found with the right methods and datasets (e.g. micro-CT^2,3^). Extrinsic hindrances, which typically concern nomenclatural issues but can also relate, for example, to the lack of availability of specimen material, are less often discussed, but can be equally difficult, or even more difficult, to overcome.

Uncertainty about allocation of scientific names is a frequent and major extrinsic hindrance that can prevent taxonomic resolution of a complex, even when some members of that complex might be diagnosable. Especially names erected before the end of the nineteenth century often pose such problems, as diagnoses and descriptions at that time were typically short and often vague, photographs in life were not available (often the taxonomists described specimens that had been sent to them already preserved by collectors, without ever having seen the organism in life), illustrations were prohibitively expensive, and specimens were preserved for their physical integrity, with no knowledge of the significance that their cells’ contents would later prove to have. With sufficient sleuthing, some such cases can be solved by narrowing down the collection locality of the specimens, identification of the current topotypical species in that area, and careful morphological examination to assign the name to one of the possibilities. This can take weeks of thorough work, can still result in an answer that is not wholly unambiguous, and is of course made considerably more challenging when the collected specimens were not accompanied with precise or accurate locality data^3–5^. These cases are common, and we need methods to deal with them with greater certainty.

Recent progress in the sequencing of DNA from museum specimens (archival DNA^6^) have the potential to completely revolutionise this process. Sequencing archival DNA is challenging because DNA degrades naturally over time, but it is made still more difficult by preservation methods; for organisms kept in ‘wet’ collections, these are often kept at room temperature in low-grade ethanol (~ 70%), which hydrolyses DNA over time. To make matters worse, formalin has often been used for preservation since its industrial production began at the end of the nineteenth century^7^. As a preservative, formalin excels, preserving morphological details very well, but unfortunately it causes damage to DNA^8^. However, some methods have been developed to partly overcome the degradation of DNA in wet specimens, including those fixed in formalin, and recover useable DNA sequences. Most of these methods combine pre-processing modifications with hybridization sequence capture^9^. Hybridization sequence capture or target enrichment uses tiled probes designed from existing genomic resources (termed ‘baits’) to fish through total genomic DNA and capture matching regions, and then sequence these using high-throughput methods^10^. Baits can be designed for a variety of purposes, from capturing restriction-associated-DNA (RAD) regions (HyRAD^11^) to targeting ultra-conserved elements (UCEs) of the genome^12^.

HyRAD and UCE-focussed approaches allow the amplification of up to tens of thousands of target markers, giving phylogenomic resolution at shallow scales particularly useful for population-level assignment (HyRAD) or very deep scales useful for highly evolutionarily distinct lineages (UCEs). However, names that need to be clarified and assigned to known lineages (or closely related unknown lineages) usually do not need this depth of resolution. Instead, they can use a smaller set of baits from the candidate lineages to directly target a few informative loci, reducing costs and increasing efficiency. Mitochondrial markers, present at much greater copy numbers than nuclear markers, are particularly useful for this purpose. Recently, we designed a set of baits that targets three mitochondrial markers (16S rRNA [16S], cytochrome oxidase I [cox1], and cytochrome b [cyt-b]) from a pooled set of Malagasy frog species^13^. This method allowed us to clarify the identity of several old names in the genus *Mantidactylus* Boulenger, 1895^14^ from Madagascar.

Here, we implement this bait set to clarify another recalcitrant name within the subgenus *Ochthomantis* Glaw & Vences, 1994^15^ of the genus *Mantidactylus*: *M. ambreensis* Mocquard, 1895. This species was described by François Mocquard at the end of the nineteenth century based on specimens from Montagne d’Ambre in northern Madagascar that had been sent to him by Charles A. Alluaud and Mr. Belly. Until recently, it was thought that the name was rather unambiguously assigned, as the species is characterised by a stark border between a brown dorsum and a white to yellow band along the flank^15–17^, a feature lacking in the other currently described *Ochthomantis* species^18^. However, we recently discovered that there are two major genetic lineages that have such a colour border on Montagne d’Ambre, one of which occurs at higher elevation and appears to be microendemic to the mountain, while the other occurs more widely across northern Madagascar at lower elevation^19^. These two species-level lineages are highly distinct in mitochondrial DNA and in all but one studied individual differ by at least one substitution in the nuclear RAG1 gene^19^. The assignment of the name being unclear, it is impossible to know which of these lineages represents the true *M. ambreensis*, and which is an unnamed species. Our results allow us to reliably assign the available name to the low-elevation, widespread lineage, and describe the high-elevation lineage as a new, micro-endemic species.

## Materials and methods

Specimens of the *Mantidactylus ambreensis* complex were collected in northern Madagascar between 1994 and 2018. All methods were carried out in accordance with the relevant guidelines and regulations. Captures were opportunistic along streams, waterfalls, and pools during diurnal and nocturnal searches. Frogs were anaesthetised by immersion in MS222 or chlorobutanol solution, and subsequently euthanised by overdose of the same substances following ethics guidelines of the American Veterinary Medical Association, and following consultation of the Animal Welfare Officer of TU Braunschweig. All field research was approved by and conducted under the guidelines of the Malagasy Ministère de l’Environnement des Eaux et des Forêts (Direction des Eaux et Forêts).

Tissue samples for molecular analysis were removed and stored separately in vials of pure ethanol, and voucher specimens then fixed in 95% ethanol and preserved in 70% ethanol. Specimens were deposited at the Zoologisches Forschungsmuseum Alexander Koenig, Bonn (ZFMK), Zoologische Staatssammlung München (ZSM), and the Mention Zoologie et Biodiversité Animale, Université d'Antananarivo (UADBA, formerly Département de Biologie Animale). Further acronyms used are FGZC, FG/MV, ZCMV, and DRV to refer to field numbers of F. Glaw, M. Vences, and D. R. Vieites; MSZC and MSTIS to field and tissue numbers of M. D. Scherz; and SRTIS to field numbers of S. M. Rasolonjatovo. The type specimen of *M. ambreensis* is preserved in the collection of the Muséum national dʼHistoire naturelle (MNHN) in Paris.

Morphometric measurements were taken by MV with the accuracy of 0.1 mm using a manual calliper and included the following variables (as used extensively in previous work on *Mantidactylus* frogs^20,21^): snout-vent length (SVL); maximum head width (HW); head length from tip of snout to posterior edge of snout opening (HL); horizontal tympanum diameter (TD); horizontal eye diameter (ED); distance between anterior edge of eye and nostril (END); distance between nostril and tip of snout (NSD); distance between both nostrils (NND); forelimb length, from limb insertion to tip of longest finger (FORL); hand length, to the tip of the longest finger (HAL); hind limb length, from the cloaca to the tip of the longest toe (HIL); foot length (FOL); foot length including tarsus (FOTL); and tibia length (TIBL). Webbing formula is given according to Blommers-Schlösser^22^ to ensure comparability with previous species descriptions of Malagasy frogs. Our description scheme (especially holotype description) is based on our previous work on the genus^20,21^.

Vocalizations were recorded in the field using a Tensai RCR-3222 tape recorder (1994) and a Marantz PMD661 MkII digital recorder outfitted with an external Sennheiser K6 + ME22 directional microphone (2016). Bioacoustic analysis followed methods outlined by Köhler et al.^23^ and used extensively by us in the past^20,24^. Recordings were sampled or re-sampled at 22.05 kHz and 32-bit resolution and computer-analysed in Adobe Audition 1.5. Frequency information was obtained through Fast Fourier Transformation (FFT; width 1024 points). Both recordings were high-pass filtered at 800 Hz to remove background noise. Spectrograms were obtained at Hanning window function with 256 bands resolution. Numerical parameters are provided as range with mean ± standard deviation in parentheses. Terminology and definitions in call descriptions follow the note-centred approach of Köhler et al.^23^. Call recordings are deposited in the Animal Sound Archive of the Museum für Naturkunde, Berlin and on FigShare (doi: 10.6084/m9.figshare.13110842).

For molecular analysis, we used sequences of fragments of the mitochondrial genes for 16S rRNA (16S) and Cytochrome Oxidase Subunit 1 (cox1) from Rasolonjatovo et al.^19^. From the comprehensive dataset published by these authors, we selected a representative set of sequences from the high elevation (HE) and low elevation (LE) lineages of *M. ambreensis*, and a sequence of the sympatric undescribed species of the subgenus *Ochthomantis*, *M.* sp. Ca63, as outgroup.

To obtain DNA sequences of the *M. ambreensis* holotype (MNHN 1893.241), we followed the ‘barcode fishing’ strategy established by Rancilhac et al.^13^: For fragments of three genes (16S, cox1, and cytochrome b), baits of 70 nucleotides in length were designed by Arbor Biosciences from sequences of the majority of Malagasy frog species (including *M. ambreensis*). After filtering based on melting temperature and collapsing 99% identical baits, 5,962 baits were retained for target enrichment. A tissue sample of thigh muscle of the *M. ambreensis* holotype was extracted from the right thigh using DNA-free scissors and stored in 100% ethanol in an Eppendorf tube filled in a lab naïve to *Mantidactylus* research. DNA extraction was performed in a clean lab dedicated to museum specimen analyses. The sample was washed with Qiagen PE Buffer, and DNA was then extracted following the protocol of Rohland et al.^25^ and purified following the protocol of Dabney et al.^26^. Library preparation was then performed using a single-stranded (ss-DNA) approach optimised for ancient and archival DNA^27,28^ using custom adapters from Gansauge and Meyer^27^ before amplification with custom Illumina indexing primers described in Paijmans et al.^29^. Optimal cycle number for amplification was previously determined using qPCR^27,30^. Subsequently, ss-DNA libraries were captured twice for the aforementioned target sequences using the Arbor Biosciences MyBaits kit. For this, we used 14.5 µL of each indexed library in a 24 h reaction at a hybridisation temperature of 65 °C. We followed the MyBaits target enrichment protocol, but reduced the bait volume to 2.75 µL and substituted the missing 2.75 µL in each reaction with nuclease-free water. After hybridization, the libraries were bound to streptavidin-coated magnetic beads, and the reactions washed and eluted according to the MyBaits kit protocol. Amplification PCR was then performed in a reaction volume of 60 µL with the following PCR conditions: 120 s @ 95 °C, then with determination of the optimal cycle number using qPCR, 30 s @ 95 °C, 45 s @ 60 °C, 45 s @ 72 °C, and final extension of 180 s @ 72 °C. Amplifications were purified using a Min Elute PCR Purification Kit (Qiagen). Final elution was in a total volume of 30 µL of 10 nM Tris–CL, 0.05% TWEEN-20 solution (pH 8.0). To increase target capture reactions success, the procedure was performed twice as described in Li et al.^31^ and Paijmans et al.^32^. Final library concentration and length distribution were determined using Qubit 2.0 and 2200 TapeStation (Aligent Technologies) assays. The enriched library was sequenced on an Illumina Next-Seq 500 sequencing platform using 500/550 High Output v2.5 (75 cycles SE, aimed at 3 million reads per sample) with custom sequencing primers^29^. After quality-trimming and adapter removal, the reads were automatically compared against reference sequences using a custom script described in Rancilhac et al.^13^, using a similarity threshold to the references of 90%, in order to reduce the data set for further analysis. Lastly, we used CodonCode Aligner 6.0.2 (CodonCode Corp.) with a majority-based alignment approach to align reads to reference sequences of *M. ambreensis* for the three gene fragments. The consensus sequences obtained from the *M. ambreensis* holotype have been submitted to GenBank (accession numbers MT982119, MT982173, and MT993842).

We aligned the holotype consensus sequences of 16S and cox1 to sequences from Rasolonjatovo et al.^19^ in MEGA v7.0^33^ (we did not further analyse the cytochrome b consensus sequence as it contained many missing data, and because reference sequences of the *M. ambreensis* HE lineage were not available for this gene). To infer phylogenetic trees, we first selected the substitution model for both genes separately under the Akaike Information Criterion in MEGA v7.0. We then inferred phylogenetic trees in MEGA v7.0 under the Maximum Likelihood (ML) optimality criterion, with SPR level 3 branch swapping and assessing node support with 1000 bootstrap replicates.

The new taxonomic name established in this work complies with the amended International Code of Zoological Nomenclature (ICZN). This article is published in an electronic journal with an ISSN (2045-2322) that is archived in PubMed Central. This publication is registered in ZooBank with the Life Science Identifier (LSID) urn:lsid:zoobank.org:pub:54957B7E-BDB3-437F-93A7-3E86288477BF (this can be resolved by appending the LSID to ‘https://zoobank.org/’).

## Results

### Identity of *Mantidactylus ambreensis*

*Mantidactylus ambreensis* was described by Mocquard^34^ based on a single specimen (the holotype) originating from Montagne dʼAmbre (without further locality information).

Illumina sequencing of this specimen (MNHN 1893.241) yielded a total of 3,659,014 reads. After selecting the reads matching a library of reference sequences (cox1 and 16S of a few *Mantidactylus* species including *M. ambreensis*) with a similarity threshold of 90%, a total of 17,847 and 290,739 reads were left for alignment, respectively (clonal reads were not removed as our goal was a majority-based assembly). Aligning these sequences against the reference sequences of both genetic lineages of *M. ambreensis* sensu lato yielded incomplete but informative consensus sequences. For the cox1 gene, 17,828 reads were aligned to the reference sequence, with a total of 214 nucleotides recovered for the total alignment length of 442 nt, with 89 nt missing at the beginning, a larger stretch of 126 missing nucleotides in the middle, and 10 nt missing at the end of the alignment; coverage for cox 1 was > 800 for the initial part (maximum 9,605), and only 4–50 for the last parts of the reconstructed sequence. For the 16S gene fragment, 289,800 reads were aligned to the reference sequence, and 371 nucleotides were recovered for an alignment of 501 nt (coverage for most stretches > 20,000, with a maximum coverage of 163,130), with 80 nt missing at the beginning, and two stretches of 32–33 and 15 nt missing in the middle of the alignment.

**Figure 1:**
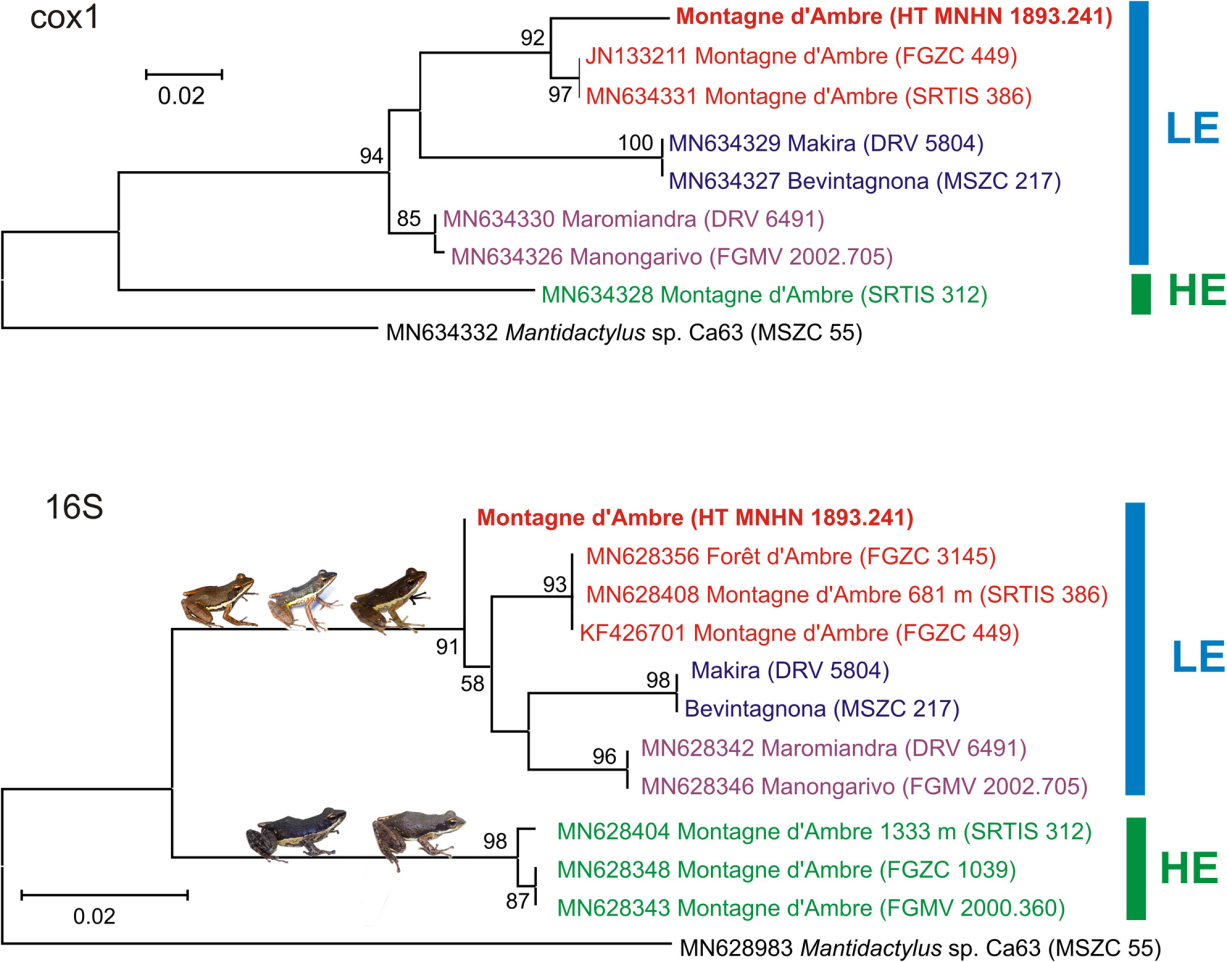
Maximum Likelihood phylogenetic trees, based on 442 and 501 nucleotide fragments of the cox1 and 16S genes (in both cases under a TN93 + G substitution model) for representative individuals of the *Mantidactylus* (*Ochthomantis*) *ambreensis* complex, with one sequence of *M.* sp. Ca63 used as outgroup. Numbers at nodes are bootstrap values in percent (shown only if > 50%) from analyses with 1000 pseudoreplicates. LE, low elevation lineage; HE, high elevation lineage. The assignment of the *M. ambreensis* holotype (HT) to LE confirms that HE corresponds to an unnamed species, described as *Mantidactylus ambony* sp. nov. herein. Colours correspond to those used by Rasolonjatovo et al.^19^. Scale bars units are substitutions per site. Inset photos show representative individuals of the two lineages.

**Figure 2:**
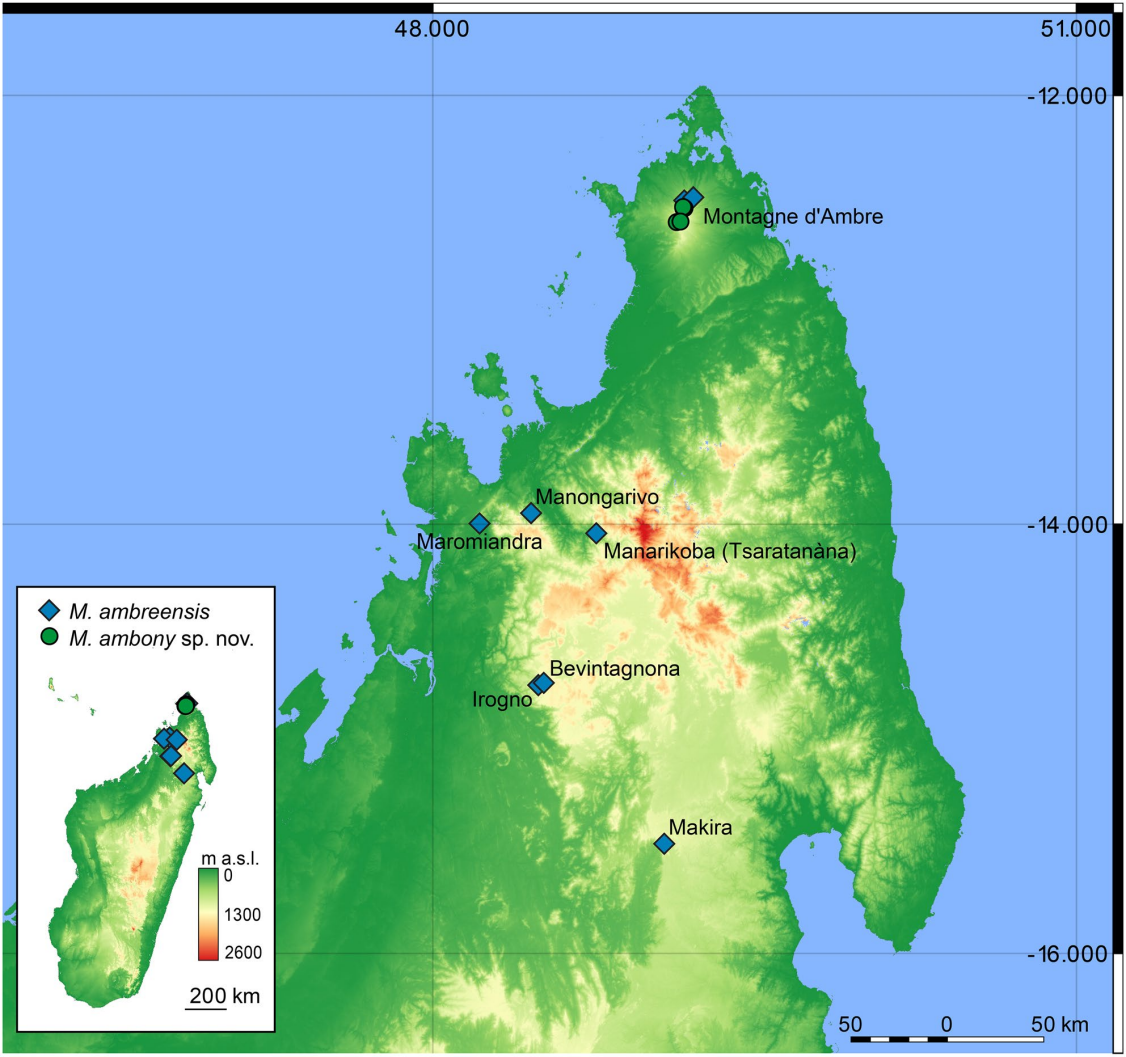
Map of northern Madagascar showing the known distribution of *Mantidactylus ambreensis* and *M. ambony* sp. nov. The base map is the USGS SRTM 1-Arc second digital elevation model. The map was created in QGIS 3.12 (available from https://www.qgis.org). Sample colours correspond to those of Fig. 1.

Phylogenetic analysis of the holotype sequences along with representative sequences selected from the data set of Rasolonjatovo et al.^19^ recovered the division of *M. ambreensis* sensu lato into two main lineages (from high elevation, HE and low elevation, LE), both of which occur on Montagne dʼAmbre with an overlapping distribution recorded around 965 m elevation above sea level (Figs. 1, 2). The holotype sequence in both gene trees was unambiguously placed in the LE lineage. The analysis of the cox1 gene fragment supported monophyly of LE (including the holotype) with a bootstrap value of 94%, and the holotype was placed with individuals from Montagne dʼAmbre (i.e., the phylogroup LE4 sensu Rasolonjatovo et al.^19^) supported by 92%. The analysis of the 16S gene fragment supported monophyly of LE including the holotype with 91%. In the tree based on this gene, the holotype was placed separately and not in the LE4 phylogroup, probably due to sequencing errors; a close inspection of lineage-diagnostic nucleotide positions in the sequences revealed 8 positions in which the holotype sequence agreed with the LE sequences, 1 in which it agreed with the HE sequences, and 1 position in which it agreed with LE sequences different from LE4 (Supplementary Fig. S1). In the cox1 sequence of the holotype, 20 diagnostic nucleotide positions suggested its belonging to the LE lineage, and none to the HE lineage (Supplementary Fig. S2).

Upon examining the morphology of the HE and LE lineages, we noted that individuals belonging to the LE lineage were larger in body size (male SVL 33.5–36.9 mm, female SVL 39.9–42.7 mm) than HE lineage individuals (male SVL 30.8–32.5 mm, female SVL 38.0 mm), and had a more pronounced supratympanic fold. The holotype of *M. ambreensis* being a female of 41.6 mm SVL, and having a faint but visible supratympanic fold (diminished by preservation and time), it also matches morphologically to the LE lineage.

Thus, we are confident in our ability to assign this taxon to the low elevation lineage that occurs from Montagne d’Ambre in the north to Makira in the south (Fig. 2). There being no available synonyms of *M. ambreensis* or any similar species, the high elevation lineage constitutes an undescribed species. In the following, we first re-describe *M. ambreensis*, and then describe the high elevation lineage as a new species.

### Re-description of *Mantidactylus (Ochthomantis) ambreensis* Mocquard, 1895

**Figure 3:**
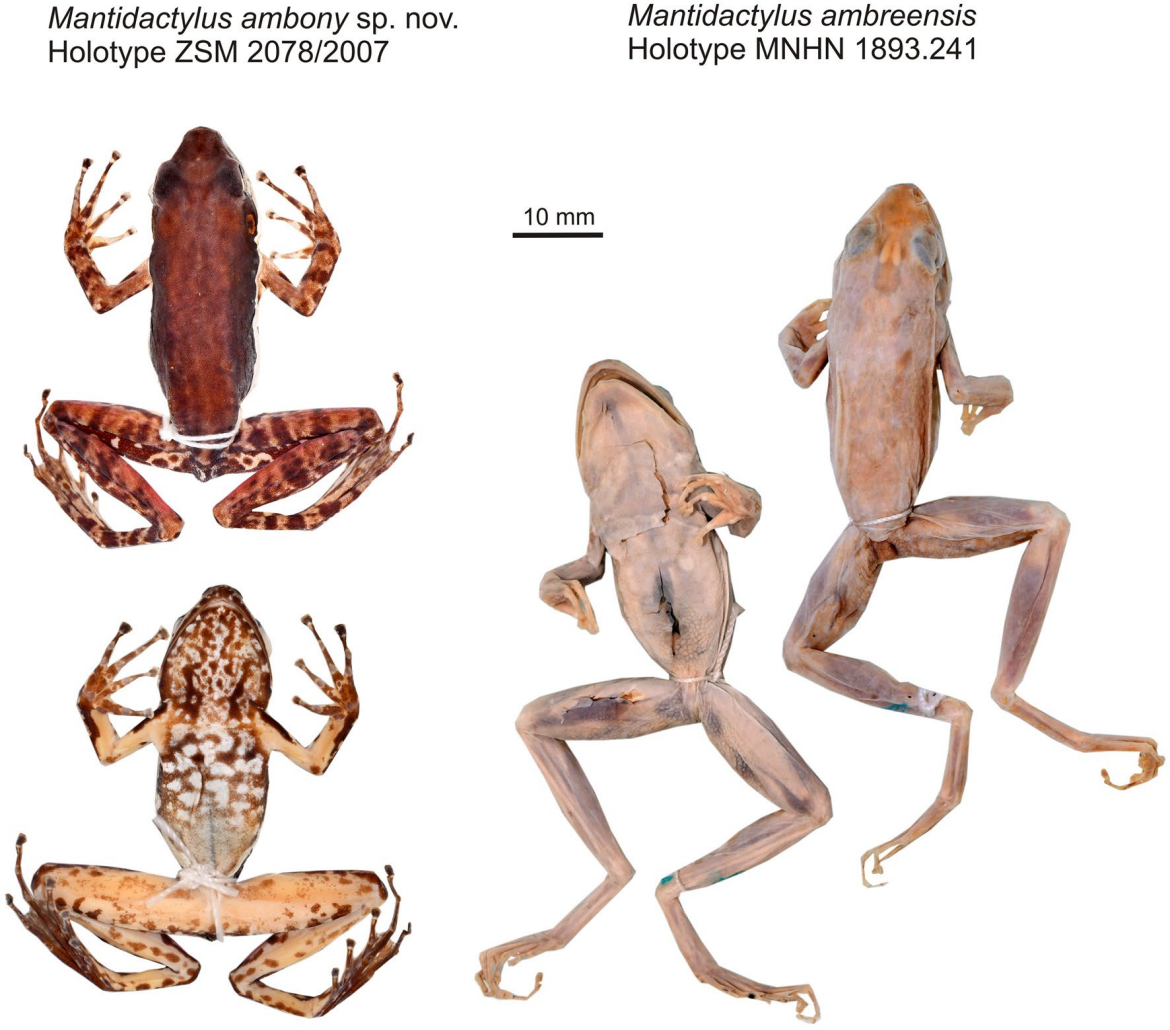
Preserved holotypes of *Mantidactylus ambony* sp. nov. (adult female, ZSM 2078/2007) and *Mantidactylus ambreensis* (adult female, MNHN 1893.241) in dorsal and ventral views.

**Figure 4:**
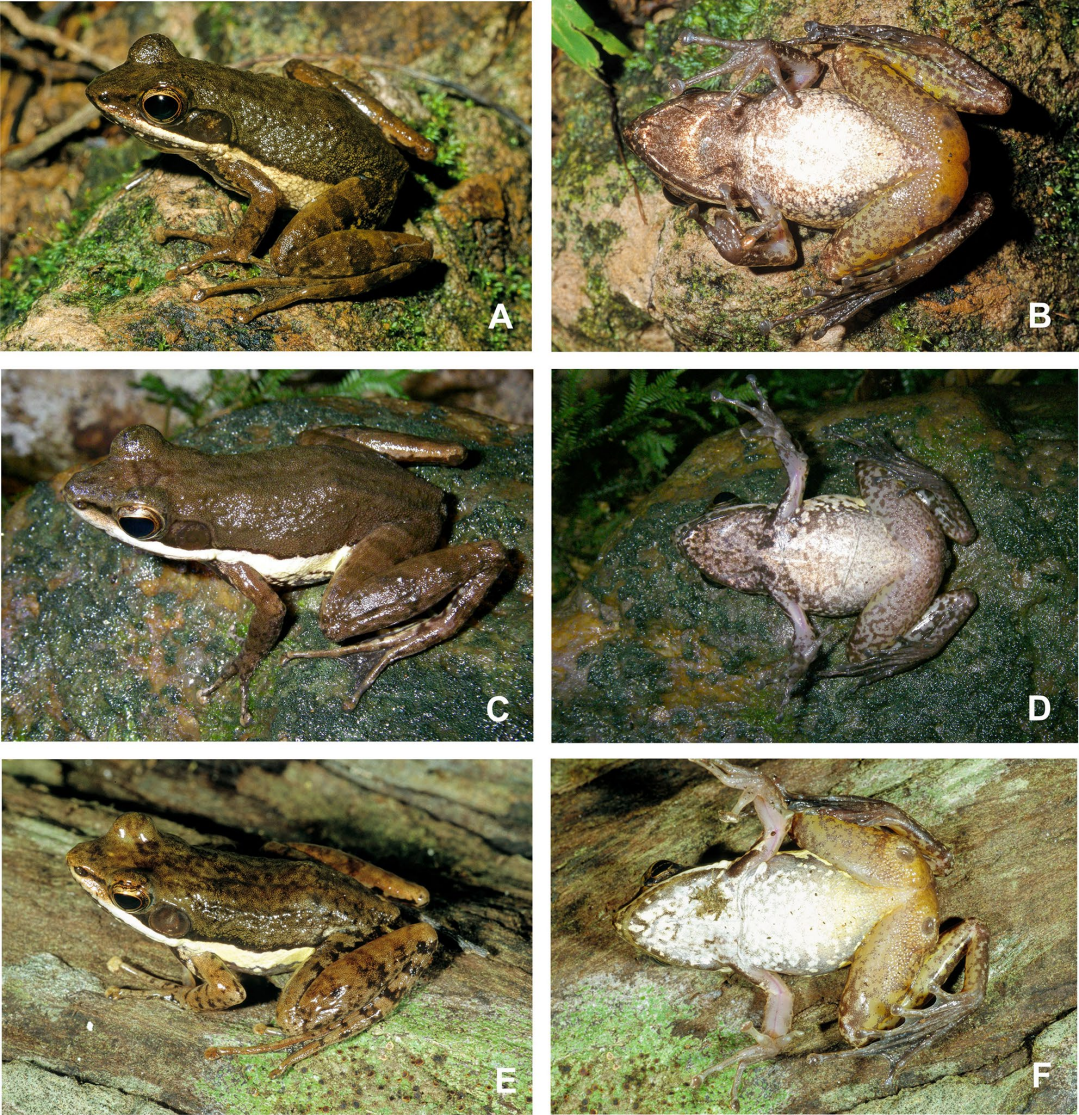
Individuals of *Mantidactylus ambreensis* in life, in dorsolateral (left) and ventral (right) views. (**A**,**B**) adult male from Andampy, Tsaratanàna (not reliably assignable to a voucher number); (**C**,**D**) adult female from the western slope of the Makira Massif (not reliably assignable to a voucher number; probably deposited in UADBA collection); (**E**,**F**) adult male from Montagne dʼAmbre, 650 m above sea level (ZSM 229/2004).

**Figure 5:**
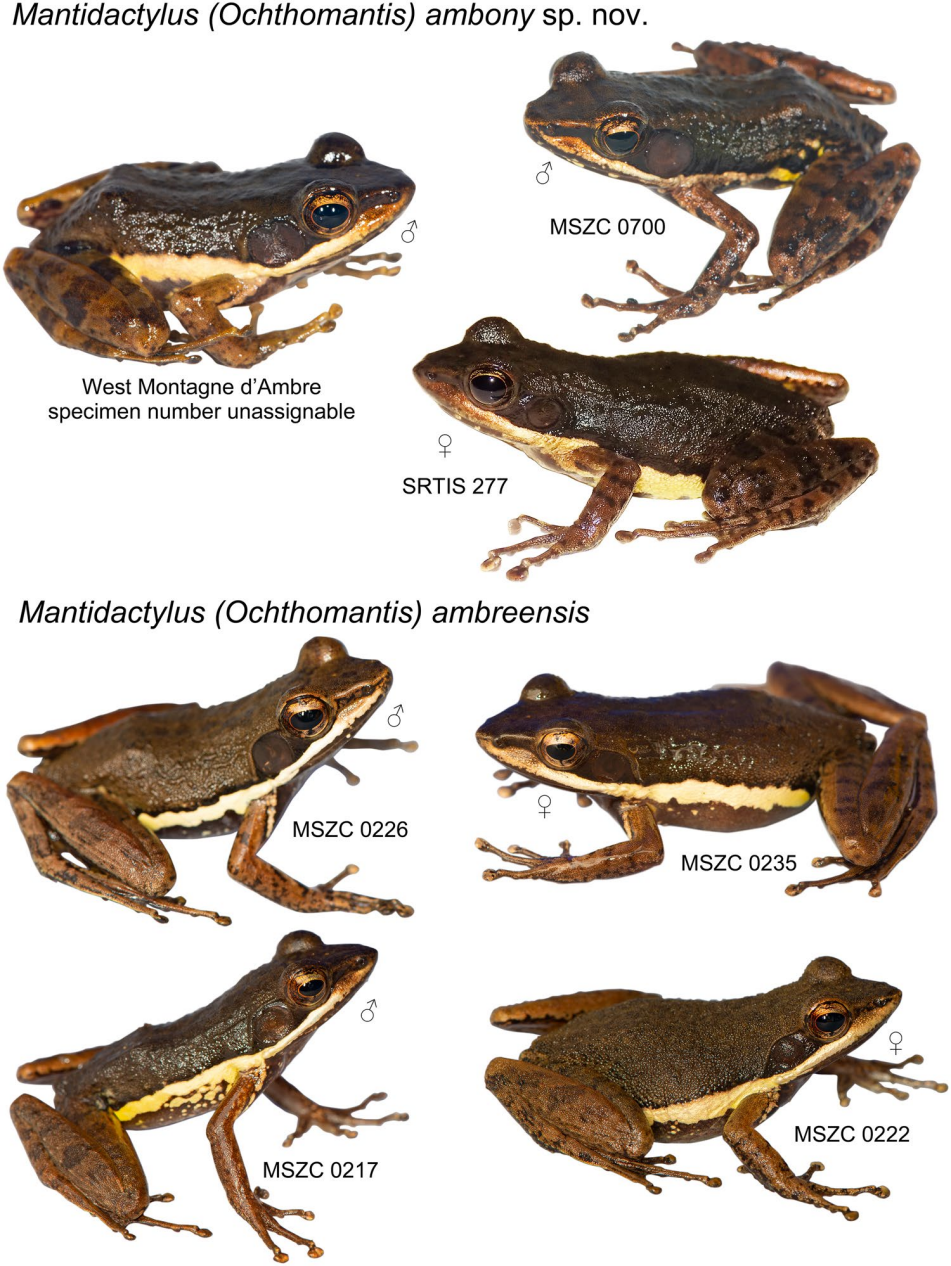
Photographs of *Mantidactylus ambreensis* and *M. ambony* sp. nov. in life (dorsolateral views). Not to scale.

**Figure 6:**
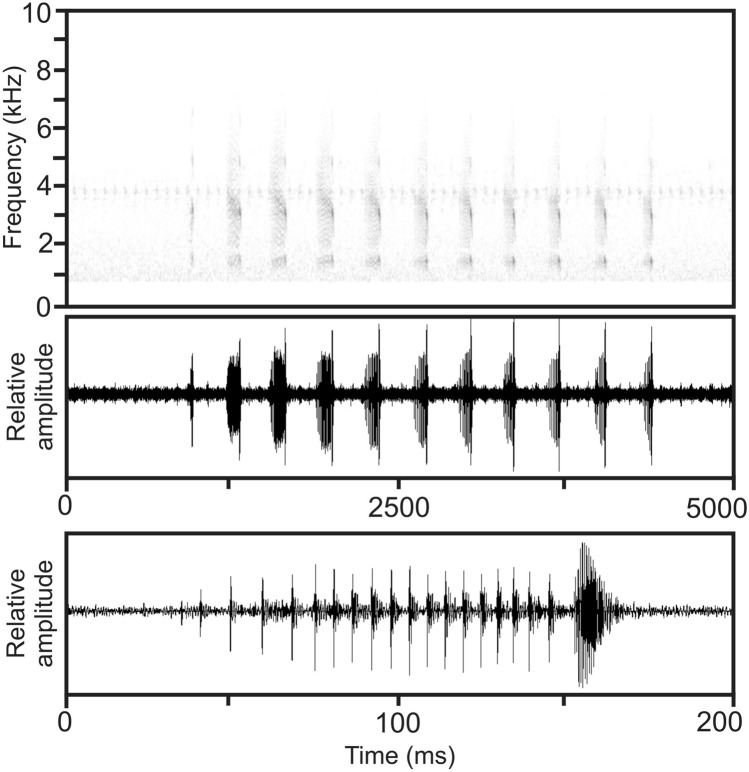
Audiospectrogram and corresponding oscillogram of an advertisement call of *Mantidactylus ambreensis* recorded at Irogno Forest on the night of 16 January 2016 (air temperature ca. 18 °C) on top. Below, an expanded oscillogram displaying the fourth note of the call. Hanning window function, FFT 256, recording high-pass filtered at 800 Hz.

Figures 3, 4, 5, 6, Table 1.

**Table 1:** Morphometric measurements (all in mm) of *Mantidactylus ambreensis* and *Mantidactylus ambony* sp. nov.

Lineage	Locality	Catalogue number	Field number	Status	Sex	SVL	HW	HL	TD	ED	END	NSD	NND	HAL	FORL	HIL	FOTL	FOL	TIBL
***Mantidactylus ambreensis***																			
LE	Montagne dʼAmbre	MNHN 1893.241		HT	F	41.6	12.2	14.2	3.8	4.7	3.7	1.4	3.4	12.3	25.6	71.2	31.6	20.0	22.5
LE1	Irogno Forest	ZSM 80/2016	MSZC 222		F	40.8	12.4	15.2	4.5	5.1	3.9	2.3	4.0	11.7	22.7	62.4	29.1	19.9	20.5
LE1	Irogno Forest	ZSM 83/2016	MSZC 235		F	41.3	12.8	14.9	3.5	5.2	3.4	2.0	3.7	11.3	23.3	64.6	30.3	19.8	21.1
LE1	Makira	ZSM 549/2009	ZCMV 11,458		F	40.9	12.7	15.0	4.3	5.9	2.2	1.7	3.2	12.6	25.6	67.5	31.2	20.7	21.7
LE2	Manongarivo	ZSM 807/2003	FG/MV 2002.704		F	42.7	13.3	16.0	4.2	5.4	4.0	2.1	3.6	11.9	26.0	69.1	31.7	20.8	22.0
LE2	Maromiandra	ZSM 561/2014	DRV 6491		F	40.9	12.7	14.9	3.6	5.0	3.1	1.8	3.4	11.6	25.1	67.8	31.8	21.3	22.1
LE3	Tsaratanàna	ZSM 634/2001	FG/MV 2001.48		F	39.9	12.6	14.3	3.1	5.0	3.5	1.9	3.3	10.3	23.2	67.6	30.0	19.2	22.3
LE4	Montagne dʼAmbre	ZSM 229/2004	FGZC 449		M	36.5	11.7	14.1	4.7	4.9	3.6	1.9	3.3	11.6	24.8	63.6	29.2	21.1	20.0
LE4	Montagne dʼAmbre	ZSM 1653/2008	FGZC 3145		M	33.5	11.8	13.4	4.7	5.2	3.4	1.7	2.7	11.5	22.8	59.1	28.5	18.9	18.5
LE1	Bevintagnona Forest	ZSM 78/2016	MSZC 217		M	34.5	11.0	13.8	4.5	4.9	3.4	1.9	3.4	11.0	22.8	60.4	27.6	18.7	18.9
LE1	Irogno Forest	ZSM 81/2016	MSZC 226		M	35.3	11.3	14.0	4.9	5.2	3.0	2.0	3.4	10.7	21.3	57.8	27.4	18.7	18.7
LE2	Manongarivo	ZSM 808/2003	FG/MV 2002.705		M	36.9	11.9	14.6	5.1	5.0	4.1	2.0	3.3	11.6	24.8	67.2	30.7	21.0	20.8
LE3	Tsaratanàna	ZSM 635/2001	FG/MV 2001.142		M	35.8	11.5	13.5	4.5	4.7	3.1	1.6	3.1	11.1	23.6	61.1	27.0	17.2	19.8
***Mantidactylus ambony sp. nov***																			
HE	Montagne dʼAmbre	ZSM 2078/2007	FGZC 1039	HT	F	38.0	12.5	13.1	3.0	4.5	3.7	2.2	3.6	12.3	26.0	66.7	29.8	20.3	20.7
HE	Montagne dʼAmbre	ZSM 492/2000	FG/MV 2000.360	PT	F (SA)	33.0	10.4	11.7	3.2	4.7	3.2	1.5	3.2	9.8	22.6	56.0	25.6	17.2	17.9
HE	Montagne dʼAmbre	ZSM 132/2018	MSZC 754	PT	M	30.8	10.0	11.6	4.1	4.1	3.1	1.6	2.7	9.7	20.8	52.0	23.8	16.4	16.0
HE	Montagne dʼAmbre	ZSM 131/2018	MSZC 633	PT	M	31.6	10.5	12.1	3.7	4.5	2.9	1.9	2.8	10.3	20.5	52.1	24.5	16.3	17.0
HE	Montagne dʼAmbre	ZSM 130/2018	MSZC 500	PT	M	32.5	10.6	12.3	5.2	5.0	3.1	1.4	2.8	10.8	22.6	57.8	25.9	17.2	17.7

For abbreviations, see “Materials and methods”.

All individuals are verified based on molecular data.

*HT* holotype, *PT* paratype, *NA* information not available, *M* male, *F* female, *SA* subadult.

#### Holotype

MNHN 1893.241, adult female, collected by Alluaud and Belly on Montagne d’Ambre between May and July 1893 (Fig. 3). The specimen is in moderately good condition despite more than a century in preservative, though rather blanched, the skin being quite translucent. A rough transverse incision was present across the pectoral girdle upon examination in 2019, connected to a parasagittal incision from the pectoral girdle toward the chin, apparently to reveal the hyoid and sternal features. A mid-ventral sagittal incision was present in the abdomen, and a further small incision has been made by us in the thigh to extract muscle tissue. The femoral glands of this specimen measure 1.6 mm long by 1.5 mm wide.

#### Additional material

ZSM 80/2016 (MSZC 0222) and ZSM 83/2016 (MSZC 0235), two adult females, and ZSM 81/2016 (MSZC 0226), an adult male, collected in forest along the Irogno river (14.7491° S, 048.4914° E, 942 m a.s.l.) on the border between the districts of Bealanana and Antsohihy, Sofia Region, at night on 16 January 2016 by M. D. Scherz and M. Rakotondratsima; ZSM 549/2009 (ZCMV 11458), an adult female, collected at Sahaovy on the western slope of Makira Reserve (15.4889° S, 049.0785° E, 607 m a.s.l.) on 20 June 2009 by M. Vences, D.R. Vieites, F.M. Ratsoavina, R.D. Randrianiaina, E. Rajeriarison, T. Rajoafiarison, and J. Patton; ZSM 807/2003 (FG/MV 2002.704), an adult female, and ZSM 808/2003 (FG/MV 2002.705), an adult male, collected at ‘Camp Norbert’ on Manongarivo (13.9481° S, 048.4578° E, 288 m a.s.l.) on 30 January 2003 by F. Glaw, R.D. Randrianiaina, and M. Vences; ZSM 561/2014 (DRV 6491), an adult female, collected in Maromiandra near Ankaramy (13.9965° S, 048.2177° E, 283 m a.s.l.) on 30 June 2010 by D. Vieites, M. Vences, R.D. Randrianiaina, S. Rasamison, A. Rakotoarison, E. Rajeriarison, and T. Rajoafiarison; ZSM 634/2001 (FG/MV 2001.48), an adult female, and ZSM 635/2001 (FG/MV 2001.142), an adult male, collected on the Tsaratanàna Massif (14.0422° S, 048.7617° E, ca. 730 m a.s.l.), on 2 February 2001 by F. Andreone, F. Mattioli, J. Randrianirina, and M. Vences at Andampy ‘Camp 0’, Manarikoba forest; ZSM 229/2004 (FGZC 449), an adult male, collected on Montagne d’Ambre (12.4900° S, 049.1719° E, ca. 650 m a.s.l.) on 22 February 2004 by F. Glaw, M. Puente, R. Randrianiaina, and A. Razafimanantsoa; ZSM 1653/2008 (FGZC 3145), an adult male, collected in the Forêt d’Ambre parcel of Montagne d’Ambre National Park (12.4753° S, 049.2142° E, 535 m a.s.l.) on 23 February 2008 by N. D’Cruze; ZSM 78/2016 (MSZC 0217), an adult male, collected in Bevintagnona (=Bivitagnono) Forest (14.7385° S, 048.5170° E, 1040 m a.s.l.) 33 km SW of Bealanana on 15 January 2016 by M.D. Scherz and M. Rakotondratsima. All localities in northern Madagascar.

#### Description

Measurements of specimens assigned to this species are presented in Table 1. A moderately sized frog, with females substantially larger than males (male SVL 33.5–36.9 mm, female SVL 39.9–42.7 mm) with smooth to bumpy skin dorsally, and smooth ventral skin (finely granular on the ventral hindlimbs). Canthus rostralis distinct, rather straight. Loreal region concave. Nostril closer to tip of snout than to eye. Tympanum distinct, 62–89% of eye diameter in females, 92–102% of eye diameter in males. Supratympanic fold distinct, running from the posterior of the eye straight posteriorly, and curving ventrally around the tympanum to the insertion of the forelimb (also faintly visible in the holotype). Lateral metatarsalia separated. Inner metatarsal tubercle distinct, outer tubercle indistinct. Finger and toe tips enlarged. Male femoral glands are oval, 6.2 mm in length and 3.3 mm in width, and they are in contact medially. Ventral colouration is mottled without clearly delimited patterns (Fig. 4B,D,F). The tympanum is darker brown than the surrounding area. The lateral white band is rather consistent, typically not interrupted with dark patches. Several live specimens are depicted in Figs. 4 and 5.

#### Larval morphology

The tadpole of this species is unknown; the species described by Randrianiaina et al.^35^ under the name ‘*M. ambreensis*’ belongs to the HE species, see below.

#### Vocalization

The advertisement call of *M. ambreensis* consists of a regular series of pulsed notes (Fig. 6). Within notes, there is significant amplitude modulation, with the terminal pulse exhibiting the highest call energy. Moreover, the terminal pulse is always substantially longer in duration compared to initial pulses of the note. Initial pulses are spaced at somewhat irregular intervals. Analysis of one call emitted by ZSM 81/2016 (MSZC 0226) recorded by M. D. Scherz on 16 January 2016 in forest along the Irogno river at an estimated air temperature of 18 °C revealed the following numerical call parameters: call duration 3487 ms, note duration 80–137 ms (109.5 ± 17.9 ms, n = 11), inter-note interval 206–254 ms (225.9 ± 15.9 ms, n = 10), notes per call 11, pulses per note 7–28 (14.4 ± 7.2, n = 11), duration of terminal pulse within notes 13–17 ms (15.2 ± 1.6 ms, n = 11), duration of initial pulses within notes all 3 ms, note repetition rate within call approximately 2.9 notes/s, pulse repetition rate within notes approximately 110–170 pulses/second, dominant frequency 1198–1543 Hz (1471 ± 136 Hz), prevalent bandwidth 1200–6400 Hz.

Before emitting these advertisement calls, the same specimen emitted a second call type different from the call that we consider to represent the advertisement call. This second call type was emitted while the individual was moving around in the vegetation. We tentatively consider this call to have a territorial function. The recording of this rather soft call is of poor quality, but it is evident that the very short notes (note duration 17–22 ms, n = 23), emitted at regular intervals (inter-note intervals 290–320 ms, n = 22, note repetition rate ca. 180 notes/minute), are composed of one single pulse only and thus lack the more complex structure evident in notes of the advertisement call.

#### Natural history

A rheophilous species that is mostly terrestrial by day but often sits on small twigs, branches, plant stems, and leaves above or near the water by night. Its rather soft advertisement calls are emitted sporadically, and often from concealed positions.

#### Etymology

A toponym referring to Montagne d’Ambre.

#### Distribution and conservation status

This species is currently known from Montagne d’Ambre, the Bealanana district, Tsaratanàna, Manongarivo, Maromiandra, and Makira (see Fig. 2; recorded elevation range 285–1040 m a.s.l.^19^), and is expected to reside in most of the intervening areas. It does not appear to occur along the northern slope of the COMATSA escarpment, as it has not been found in Sorata, Andravory, or Marojejy. It should be noted that the record from Maevatanana in central western Madagascar that is included in the species’ IUCN Red List record, 10.2305/IUCN.UK.2016-3.RLTS.T57459A84169081.en, is of uncertain provenance, and we consider it highly unlikely that it refers to *M. ambreensis*. This species thus evidently has a wide distribution. However, low elevation forest in Madagascar is under particular threat from deforestation; on the border of the Bealanana District (Irogno forest), this species is persisting in diminutive fragments of riparian forest, but the stability of these populations is questionable. Thus, the spread of risk to the species is great, but each population across its distribution is likely highly threatened, despite in many cases being within Madagascar’s network of protected areas^36^. A minimum convex polygon encompassing known locations has an approximate area of 22,000 km^2^. At present it does not therefore qualify as threatened under criterion B of the IUCN Red List^37^, as its extent of occurrence is > 20,000 km^2^, and it is likely to occur at more than 10 locations. We conservatively propose therefore to continue to consider it as Least Concern in the IUCN Red List, following our recent re-assessment of it, but emphasise that the point from Maevatanana should be removed.

## Description of a new species

### *Mantidactylus (Ochthomantis)ambony* sp. nov

LSID: urn:lsid:zoobank.org:act:2F3F14D1-6D0E-49E5-9646-8167E64CFDF3.

**Figure 7:**
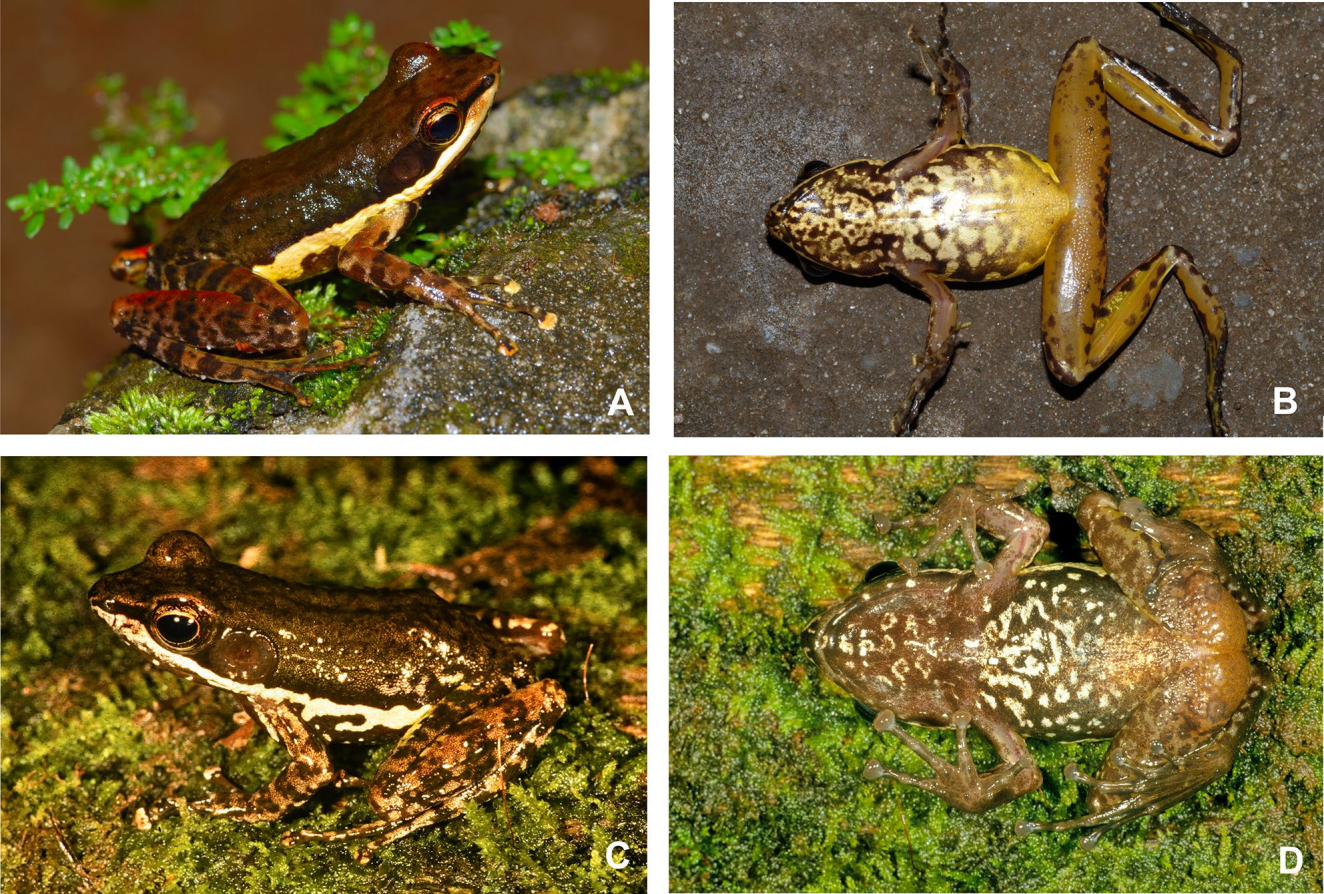
Individuals of *Mantidactylus ambony* sp. nov. from Montagne dʼAmbre in life, in dorsolateral (left) and ventral (right) views. (**A**,**B**) female holotype (ZSM 2078/2007); (**C**,**D**) adult male paratype (ZFMK 57417, call voucher).

**Figure 8:**
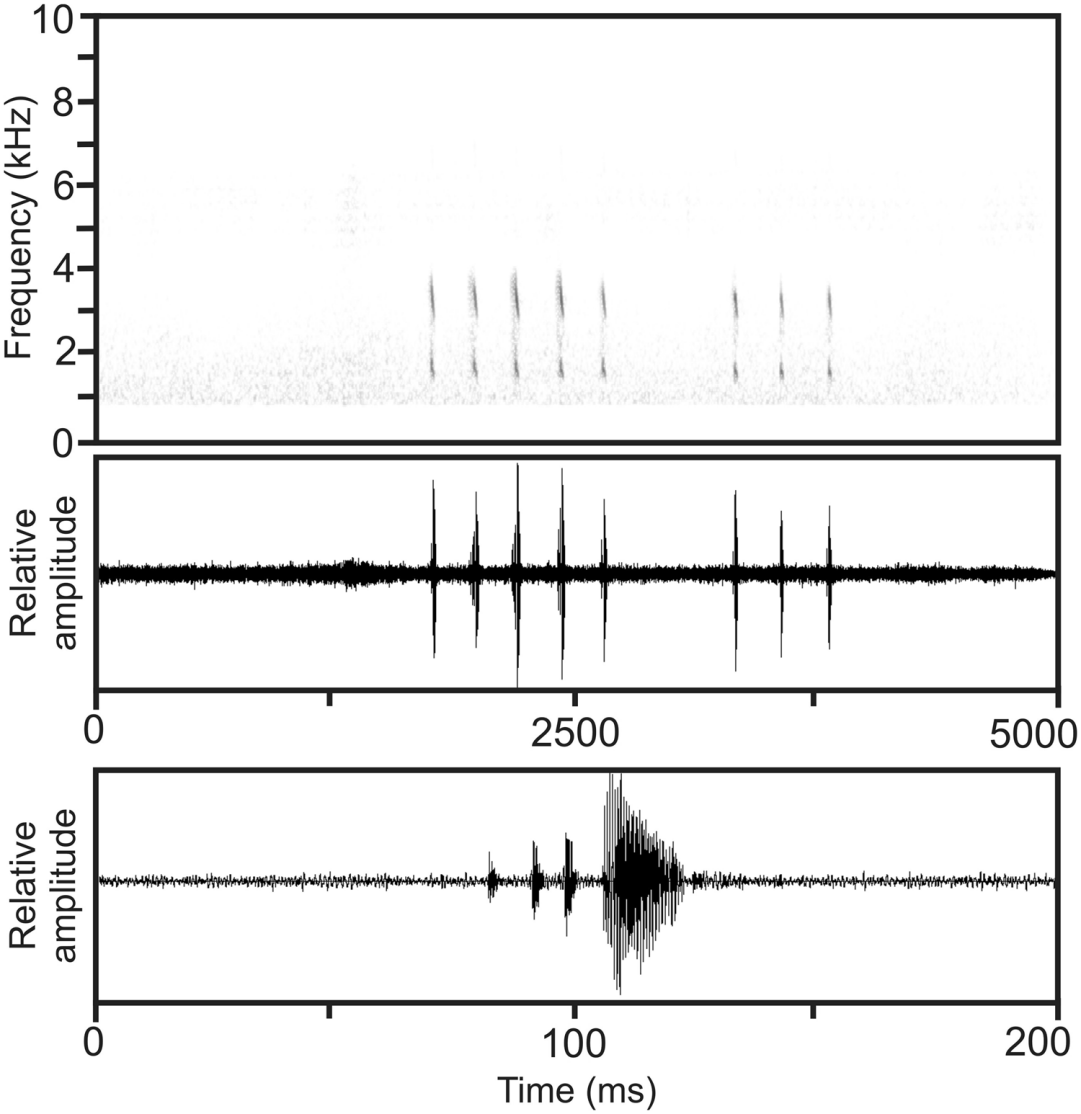
Audiospectrogram and corresponding oscillogram of two advertisement calls of *Mantidactylus ambony* sp. nov. recorded on 15 March 1994 on Montagne d’Ambre (air temperature 21 °C) on top. Below, an expanded oscillogram displaying the third note of the first call. Hanning window function, FFT 256, recording high-pass filtered at 800 Hz.

Figures 3, 7, 5, 8, Table 1.

#### Holotype

ZSM 2078/2007 (FGZC 1039), adult female, from the vicinity of the Gîte d’Étape in Montagne dʼAmbre National Park (12.5280° S, 049.1720° E, ca. 1050 m a.s.l.), northern Madagascar, collected at night on 24 February 2007 by F. Glaw, P. Bora, H. Enting, J. Köhler, and A. Knoll (Fig. 3).

#### Paratypes

ZSM 130/2018 (MSZC 0500/SRTIS 46), adult male, collected at mid-elevation on Montagne d’Ambre (12.5257° S, 049.1729° E, 1050 m a.s.l.) at night on 15 November 2017 by M.D. Scherz, J.H. Razafindraibe, A. Razafimanantsoa, O. Randriamalala, S.M. Rasolonjatovo, R. Tiavina, and A. Rakotoarison—identification verified using Illumina DNA barcode sequencing; ZSM 131/2018 (MSZC 0633/SRTIS 72), adult male, collected at mid-elevation on Montagne d’Ambre (12.5209° S, 049.1666° E, 1080 m a.s.l.) at 18h19 on 17 November 2017 by M.D. Scherz, J.H. Razafindraibe, A. Razafimanantsoa, O. Randriamalala, S.M. Rasolonjatovo, R. Tiavina, and A. Rakotoarison; ZSM 132/2018 (MSZC 0754/SRTIS 193), adult male, collected on the western slope of Montagne d’Ambre (12.5910° S, 049.1366° E, 966 m a.s.l.) at 20h11 on 7 December 2017 by M.D. Scherz, J.H. Razafindraibe, A. Razafimanantsoa, and S.M. Rasolonjatovo; ZSM 492/2000 (FG/MV 2000.360), a subadult female, collected at ca. 1000 m a.s.l. on Montagne d’Ambre (12.517° S, 49.167° E) on 18 March 2000 by F. Glaw, K. Schmidt, and M. Vences; ZFMK 57417, adult male (call voucher), from Montagne d’Ambre, collected by F. Glaw, N. Rabibisoa, and L. Ramilison on 15 March 1994; UADBA uncatalogued (MSZC 0462/SRTIS 27), adult male, collected at mid-elevation on Montagne d’Ambre (12.5270° S, 049.1677° E, 1096 m a.s.l.) at 12h50 on 14 November 2017 by M.D. Scherz, J.H. Razafindraibe, A. Razafimanantsoa, O. Randriamalala, S.M. Rasolonjatovo, R. Tiavina, F.M. Ratsoavina, Z. Rakotomalala, and A. Rakotoarison; UADBA uncatalogued (MSZC 0499/SRTIS 60), adult male, collected at mid-elevation on Montagne d’Ambre (12.5257° S, 049.1730° E, 1036 m a.s.l.) at night on 15 November 2017 by M.D. Scherz, J.H. Razafindraibe, A. Razafimanantsoa, O. Randriamalala, S.M. Rasolonjatovo, R. Tiavina, and A. Rakotoarison; UADBA uncatalogued (MSZC 0632/SRTIS 67), adult female, collected at mid-elevation on Montagne d’Ambre (12.5208° S, 049.1663° E, 1087 m a.s.l.) at 15h52 on 17 November 2017 by M.D. Scherz, J.H. Razafindraibe, A. Razafimanantsoa, O. Randriamalala, S.M. Rasolonjatovo, R. Tiavina, and A. Rakotoarison; UADBA uncatalogued (MSZC 0700), adult male, collected at high elevation near Lac Maudit on Montagne d’Ambre (12.5884° S, 049.1542° E, 1274 m a.s.l.) at 21h29 on 26 November 2017 by M.D. Scherz, J.H. Razafindraibe, A. Razafimanantsoa, O. Randriamalala, S.M. Rasolonjatovo, R. Tiavina, and A. Rakotoarison. All localities in northern Madagascar.

#### Additional material

ZSM 762/2004 (FG/MV 2002.1950), a tadpole at Gosner stage 25, collected in a brook in Montagne d’Ambre National Park with a max depth 15 cm, width 30 cm, on 17 February 2003.

#### Diagnosis

The new species is assigned to the genus *Mantidactylus* based on the presence of an intercalary element between terminal and subterminal phalanges of fingers and toes (verified by external observation), and of a central depression in femoral glands, presence of a rudimentary femoral gland in the female, tympanum diameter distinctly larger in males compared to females, and molecular phylogenetic relationships. Within *Mantidactylus*, it is assigned to the subgenus *Ochthomantis* by the combination of (1) moderate body size (male SVL 30.8–32.5 mm, female SVL 38 mm), (2) absence of dorsolateral colour border, (3) presence of a distinct frenal stripe, (4) presence of large yellowish to whitish patches or stripes in the inguinal region or between colouration of flanks and belly, (5) moderately webbed feet, (6) enlarged finger and toe pads, (7) presence of vomerine teeth, (8) slightly distensible single subgular vocal sac in males, (9) soft calls, (10) riparian habits, living close to the water in streams, (11) presence of reniform dark markings on the posterior throat (in most specimens), (12) tadpoles with reduced jaw sheaths and keratodonts, and (13) molecular phylogenetic relationships (see Glaw and Vences^38^).

Within *Ochthomantis*, the new species is characterized by the unique combination of the following features: relatively small size (male SVL 30.8–32.5 mm, female SVL 38 mm), an indistinct supratympanic fold, presence of a sharply delimited continuous white to yellow lateral band separating the dorsolateral from the ventral colouration, distinct until beneath the eye and sometimes also reaching the tip of the snout, and usually marbled white and brown ventral colouration.

The species can be distinguished from *M. femoralis* by smaller body size (male SVL 30.8–32.5 mm vs up to 37.2 mm, female SVL 38.0 mm vs 44.7–53.5 mm) and by presence of a distinct white lateral stripe (vs an oblique, generally short inguinal patch of usually yellow colouration); from *M. mocquardi* by substantially smaller body size (male SVL 30.8–32.5 mm vs up to 41.0 mm, female SVL 38.0 mm vs 62.9 mm) and by presence of a distinct white lateral stripe (vs absence); from *M. majori* by substantially smaller body size (male SVL 30.8–32.5 mm vs up to 38.7 mm, female SVL 38.0 mm vs 44.8 mm), by presence of a distinct white lateral stripe (vs sharp border to the relatively uniform whitish venter), by less extensive webbing (*M. majori* has almost fully webbed feet), and by substantially different tadpole morphology; from *M. zolitschka* by slightly larger body size (male SVL 30.8–32.5 mm vs up to 28.8–30.6 mm, female SVL 38.0 mm vs 33.6–37.7 mm) and by presence of a distinct white lateral stripe (vs a small yellowish inguinal patch) (note: size information of these species is based on measurements of Glaw and Vences^17^ based mostly on type material).

The new species is sister to *M. ambreensis*, from which it differs by numerous nucleotide substitutions in various mitochondrial genes; in almost all individuals by at least one substitution in the nuclear RAG1 gene; by smaller body size (male SVL 30.8–32.5 vs 33.5–36.9, female SVL 38.0 vs 39.9–42.7); by a less distinct supratympanic fold, and the following details of their advertisement calls: substantially higher note repetition rate (4.5 vs 2.9 notes/second), substantially lower number of pulses per note (3–5 vs 7–28), and shorter note duration (29–63 vs 80–137 ms). Given that calls of *M. ambreensis* were recorded at only slightly lower temperature, the differences in calls are far beyond of what can be expected to represent intra-specific variation (see Köhler et al.^23^).

In addition *M. ambony* sp. nov. is separated from all other species in the subgenus *Ochthomantis* by numerous substitutions in the 16S rRNA mitochondrial marker, amounting to an uncorrected pairwise distance of 5.6–6.8%^19^.

#### Description of the holotype

ZSM 2078/2007 (FGZC 1039), an adult female in a very good state of preservation. Measurements are given in Table 1. Body slender. Head longer than wide, as wide as body. Snout long and subacuminate in dorsal and lateral view. Nostrils directed laterally, slightly protuberant, much nearer to the tip of the snout than to the eye. Canthus rostralis distinct, slightly concave, loreal region concave. Tympanum distinct, smaller than eye, oval, horizontal diameter of tympanum 67% of horizontal eye diameter. Supratympanic fold indistinct. Tongue removed as tissue sample. Maxillary teeth present. Vomerine teeth form two rounded aggregations, positioned posterolateral to choanae. Choanae rounded. Subarticular tubercles single. Outer and inner metacarpal tubercle distinct. Fingers without webbing. Relative length of fingers: I < II < IV < III. Finger discs moderately enlarged. Nuptial pads absent. Foot slightly shorter than tibia (98%). Lateral metatarsalia separated. Inner metatarsal tubercle present, outer metatarsal tubercle small but distinct. Webbing formula: 1(0.5), 2i(1), 2e(0.25), 3i(1.5), 3e(1), 4i(2), 4e(2), 5(0.5). Relative length of toes: I < II < III < V < IV, third toe slightly shorter than fifth. Skin on the upper surface smooth. Ventral side smooth. Femoral glands tiny and brown, difficult to distinguish from the patterning of the ventral thigh.

Colour in preservative (after 13 years in preservative) dorsally uniform brown, extending to the mid flank. Colour border along the flank is stark between the dorsal colouration and the white lateral line, which runs from the inguinal region to the tip of the snout, though it fades anterior to the eye. The colour border is dorsally rather straight, but ventrally more reticulated, and is a narrow stripe so that the insertion of the arm is not engulfed. The tympanic region is slightly darkened. The dorsal forelimbs and hindlimbs are mottled dark and light brown, the first two fingers and parts of the toes having also a cream colouration. The hidden surfaces of the dorsal thighs are also lighter in base colour. There are light annuli before each terminal disc. The ventral colouration is yellow cream on the limbs, and more whitish on the abdomen and chin. It is marbled on the trunk with brown, and the reniform markings on the pectoral girdle and posterior chin are distinctly darker than the other browns of the venter. The ventral limbs are less mottled, with occasional spots of brown. The hands and feet are ventrally brown. Colouration in life can be seen in Fig. 7.

#### Variation

Measurements of specimens assigned to this species are presented in Table 1. Variation in colouration in life is shown in Figs. 7 and 5. ZSM 132/2018 has the third and fifth toe of equal length. The only verified adult female (the holotype, ZSM 2078/2007) is substantially larger than our male specimens (38.0 mm vs 30.8–32.5 mm, respectively). The tympanum of males is larger than in the single female (TD 80–104% of ED vs 67%). Males have much larger and more distinct femoral glands, of type 3 with a distinct central opening^39^ in their main part which (ZSM 131/2018) is circular, with a diameter of 3.5 mm. The glands are centrally almost in contact with one another. In the males, the ventral side can vary from uniform dark with little fine whitish ventral spotting, to more distinctly marbled patterning. In all of the males, the lateral band contains some dark markings. The tympanic region is dark brown.

#### Larval morphology

The tadpole of this species was described by Randrianiaina et al.^35^ under the name ‘*M. ambreensis*’.

#### Vocalization

The advertisement call of *M. ambony* sp. nov. consists of a series of pulsed notes repeated at regular intervals (Fig. 8). Within notes, there is significant amplitude modulation, with the terminal pulse exhibiting the highest call energy. Moreover, the terminal pulse is always significantly longer in duration compared to initial pulses of the note. Analysis of four calls emitted by ZFMK 57417 (31.0 mm SVL; Fig. 7) recorded by F. Glaw on 15 March 1994 on Montagne d’Ambre at 21 °C air temperature (Vences et al.^40^, CD2, track 91) revealed the following numerical call parameters: call duration 518–1656 ms (951.8 ± 497.1 ms, n = 4), note duration 29–63 ms (43.2 ± 10.2 ms, n = 4), inter-note interval 158–191 ms (172.7 ± 10.8 ms), notes per call 3–8 (5.0 ± 2.2, n = 4), pulses per note 3–5 (3.8 ± 0.8), duration of terminal pulse within notes 14–17 ms (15.8 ± 1.1 ms, n = 4), duration of initial pulses within notes all 1–2 ms, note repetition rate within calls approximately 4.5 notes/second, pulse repetition rate within notes approximately 160 pulses/second, dominant frequency 1538–1755 Hz (1646 ± 49 Hz), prevalent bandwidth 1200–4000 Hz.

#### Natural history

As for *M. ambreensis*, this species is rheophilous and mostly terrestrial by day, sitting on the ground, on rocks, wood, lichen, or hiding under rocks, always in close proximity to streams. The species is semi-arboreal by night, often found on elevated spots above the water level (once recorded at up to 2 m height), and sitting on substrates like leaves, rocks, dead wood, and plant stems. Its soft calls are emitted sporadically, and often from rather concealed positions.

#### Etymology

The species epithet ‘ambony’ (pronounced am-BOO-nee) is a Malagasy word meaning ‘above’, in reference to this species’ distribution at higher elevation compared to that of *M. ambreensis*. It is used as an invariable noun in apposition.

#### Distribution and conservation status

*Mantidactylus ambony* sp. nov. is apparently microendemic to mid- to high-elevation areas of Montagne d’Ambre (recorded elevational range 940–1375 m a. s. l.^19^). In the absence of current or immediate future threats to habitat integrity we suggest an IUCN conservation status of Near Threatened for this species despite its small extent of occurrence, as we have recently proposed for syntopic *Stumpffia* species^41^.

## Discussion

The revelation that ‘*Mantidactylus ambreensis*’ contained two deep genetic lineages^19^ was surprising, as no cryptic lineages had previously been detected in this complex, although a great deal of cryptic diversity is known from the related *M. femoralis/mocquardi* species complex^35,42^. Rasolonjatovo et al.^19^ showed that these lineages have almost entirely exclusive nuclear RAG1 gene haplotypes (one individual of the low elevation lineage from Irogno (Bealanana District) shared one of the high elevation lineage haplotypes present on Montagne d’Ambre), as well as strong mitochondrial divergence (5.6–6.8% 16S rRNA divergence, much greater than the typical 3% threshold for amphibians^43^). Furthermore, one specimen of the low-elevation lineage showed genome-wide differentiation in a large number of the 5065 nuclear markers obtained by FrogCap target enrichment^19^, and the two lineages formed two separate clusters without apparent admixture in an analysis of microsatellites (data to be published elsewhere). Prior to that study, we had not suspected species-level divergence in this group, but this mito-nuclear concordance and the level of genetic differentiation made it clear that these were very likely two distinct species. Yet, with this data in hand, there remained both intrinsic hindrances to the resolution of the taxonomy of the group (morphologically cryptic lineages) and the extrinsic hindrance of the uncertainty of assignment of the name *M. ambreensis*. Using an integrative dataset of bioacoustics and morphology, we were able to overcome the intrinsic hindrance, identifying subtle differences between the highland and lowland forms in body size, morphology, colouration, and distinct differences in the advertisement calls. The extrinsic hindrance we overcame by using our bait set for Malagasy frogs to determine that the holotype of *M. ambreensis* belongs to the lowland lineage, which is actually widespread across northern Madagascar. This being clarified, we were able to describe the new species *M. ambony*, which is microendemic to mid- to high-elevation sites of Montagne d’Ambre. The two species appear to be largely elevationally segregated, but may occur in sympatry in a contact zone around 950–1050 m a.s.l. on Montagne d’Ambre.

The discovery of this new species and the resulting taxonomic changes have implications for both biogeography and conservation. The biogeographic implications (discussed in greater detail in Rasolonjatovo et al.^19^) are that these two species did not speciate in situ, as might be expected from their ecological differences (as evidenced by segregated elevational distribution), but instead suggests a hypothetical scenario of vicariant speciation in different refugia, with subsequent expansion of the low elevation lineage and re-colonisation of the lowland forests of Montagne d’Ambre. From a conservation perspective, this means that *M. ambreensis* is in fact a widespread species that also survives in forests that are heavily degraded and fragmented, whereas *M. ambony* is microendemic to Montagne d’Ambre. These insights emphasise the importance of improved taxonomic understanding for conservation considerations^21,44^.

The method we have implemented here and in Rancilhac et al.^13^ that we term ‘barcode fishing’ shares some methodological similarities (e.g. use of single-stranded libraries following Gansauge et al.^28^) with the recent study by Evans et al.^5^ aimed at achieving a similar goal, but the two approaches differ conceptually. The method used by Evans et al.^5^ involves sequencing of the nearly complete mitochondrial genome of archival specimens, using specifically designed baits. In contrast, our method targets specifically the three mitochondrial genes for which we have the most complete reference database of related frogs. In comparison to the method of Evans et al.^5^, our method yields far fewer basepairs of sequence once consensuses are collapsed, but makes up for this by maximising read depth on the targeted regions, and being designed for broad phylogenetic implementation; it can be applied to any of the organisms used in probe design without modification—in our case all of the frogs of Madagascar.

So far, we have applied our Malagasy frog ‘barcode fishing’ bait set only to small groups of frogs^13^ (including the present paper), rather than to large species complexes, where intrinsic and extrinsic hindrances are compounded by diversity. The obvious next step is to use the same approach for dealing with larger species complexes. One particularly troublesome clade, *Brygoomantis* Dubois, 1992, a subgenus of *Mantidactylus* that currently contains 12 nominal species, at least five synonyms, but also 23 candidate species^43,45^, will make a perfect candidate for the exploration of this approach in a large complex, and will doubtless result not only in numerous new species^20^ but also in the resurrection of several old names.

Similar ‘barcode fishing’ probe sets will doubtless prove highly useful in other areas with relatively well-characterised biota, where intrinsic hindrances to resolution can be alleviated with an integrative approach, but extrinsic hindrances persist. Moreover, they can draw on the huge success of DNA barcoding initiatives^46–48^ to develop baits with which to target type material. These novel technical possibilities present an exciting future where extrinsic hindrances such as nomenclatural issues will be overcome with comparative ease. However, it will also be crucial to establish best-practice guidelines and carefully evaluate the costs and benefits of applying such methods at the grand scale. Firstly, it must be remembered that analyses of archival DNA are not error free—for instance, in our case we hypothesise that the 16S rRNA sequence recovered from the *M. ambreensis* holotype may contain a few sequencing errors that led to its placement outside the LE4 haplogroup in a phylogenetic analysis of that gene, contrary to the cox1 tree (Fig. 1; Supplementary Fig. S1). The method is also susceptible to contamination, especially if laboratory procedures are not carried out in a dedicated aDNA laboratory as in our study, and this could lead to apparently reliable molecular assignment of type material that is actually wrong. Lastly, it should be kept in mind that this approach has the potential to destabilise names that have not been in question for centuries if it is found that they have been misapplied, which is doubtless the case for a small but substantial number of the approximately 1.8 million named eukaryote species. We suggest that taxonomists should primarily devote their resources to exploring the biosphere and naming the many millions of new species that remain undescribed. In this ambitious task, targeted sequence capture to clarify the identity of historical types can become a crucial component—it will alleviate hindrances that block taxonomic progress, like the example herein, and help us to overcome these important barriers between current knowledge and the completion of the inventory of life on Earth.

## Supplementary information

Supplementary Information.
