# Supplementary material for: ‘Barcode fishing’ for archival DNA from historical type material overcomes taxonomic hurdles, enabling the description of a new frog species
Source: Sci Rep. 2020 Nov 5;10:19109. doi: 10.1038/s41598-020-75431-9 (PMC7644772; doi:10.1038/s41598-020-75431-9)
Supplement: Supplementary file 1 — Supplementary Information. [file 41598_2020_75431_MOESM1_ESM.pdf]

Supplementary figures to

‘Barcode fishing’ for archival DNA from historical type material overcomes taxonomic hurdles, enabling the description of a new frog species

Mark D. Scherz[1,2,3], Safidy M. Rasolonjatovo[4,5], Jörn Köhler[6], Loïs Rancilhac[1], Andolalao Rakotoarison[4,7], Achille P. Raselimanana[4,5], Annemarie Ohler[8], Michaela Preick[9], Michael Hofreiter[9], Frank Glaw[2], Miguel Vences[1*]

[1]Zoologisches Institut, Technische Universität Braunschweig, Mendelssohnstr. 4, 38106 Braunschweig, Germany

[2]Zoologische Staatssammlung München (ZSM-SNSB), Münchhausenstr. 21, 81247 München, Germany

[3]Current address: Department of Evolutionary Biology, Universität Konstanz, Universitätsstr. 10, 78464 Konstanz, Germany

[4]Mention Zoologie et Biodiversité Animale, Université d’Antananarivo, BP 906, 101 Antananarivo, Madagascar

[5]Association Vahatra, BP 3972, Lot V A 38 LBA Ter Ambohidempona Tsiadana, Antananarivo 101, Madagascar

[6]Hessisches Landesmuseum Darmstadt, Friedensplatz 1, 64283 Darmstadt, Germany

[7]School for International Training, VN 41A Bis Ankazolava Ambohitsoa, 101 Antananarivo, Madagascar

[8]Museum national d’Histoire naturelle, ISYEB, CNRS, SU, EPHE, UA, 57 Rue Cuvier, 75005 Paris, France

[9]Institut für Biochemie und Biologie, Universität Potsdam, Karl-Liebknecht-Str. 24-25, 14476 Potsdam, Germany

*Corresponding author: Miguel Vences, m.vences@tu-braunschweig.de

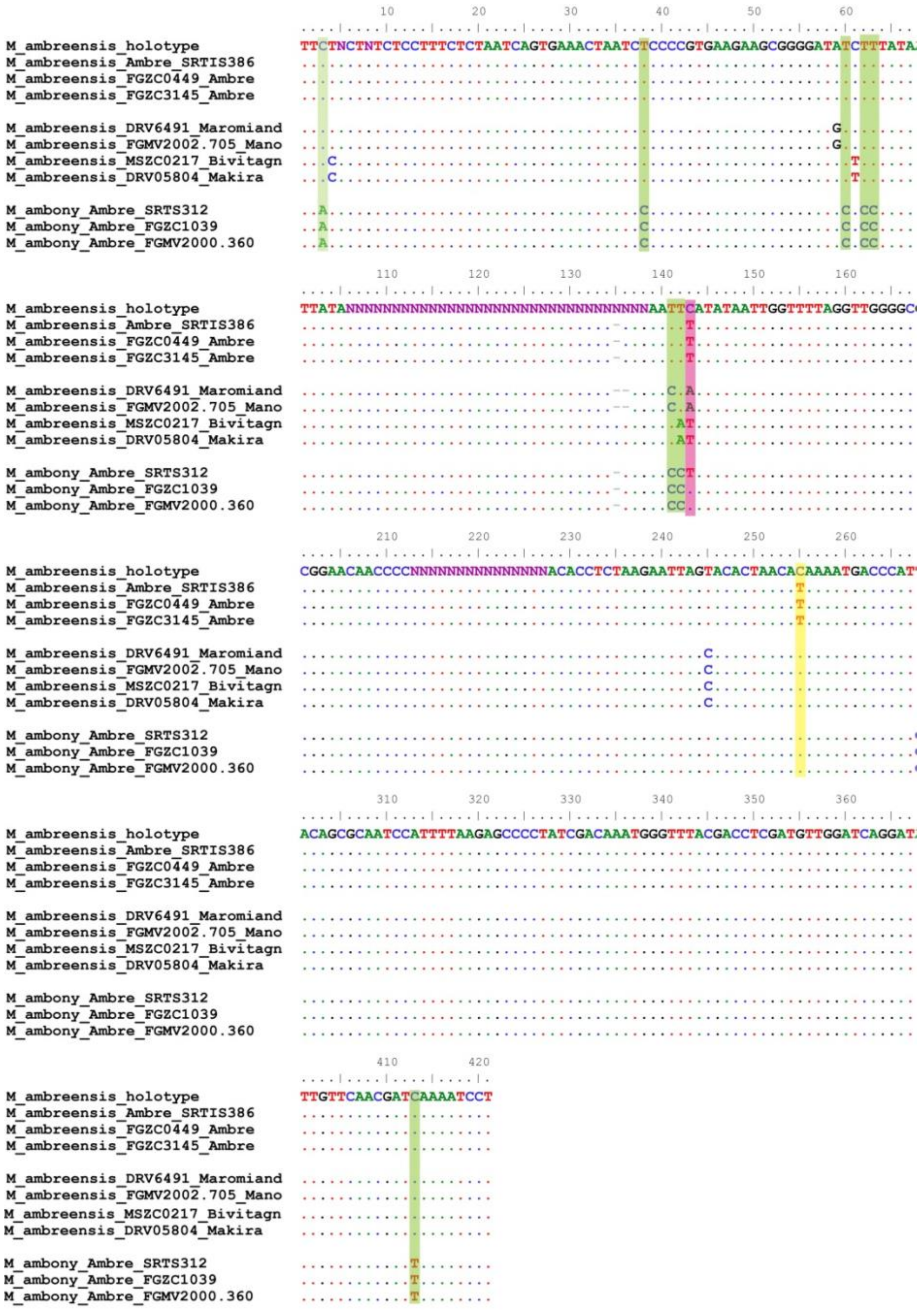

Supplementary Figure S1. Alignment of the 16S rRNA gene, showing differences of the reconstructed sequence of the *Mantidactylus ambreensis* holotype (obtained by targeted enrichment sequencing) in comparison with selected sequences of specimens obtained by Sanger sequencing. Green bars highlight eight diagnostic alignment positions in which the holotype agrees with specimens of the LE lineage but differs from the HE lineage (that is herein described herein as *M. ambony* sp. nov.). A red bar marks the single position in which the holotype agrees instead with the HE lineage, and a yellow bar marks a position in which the holotype agrees with the HE lineage, but also with non-topotypical specimens of the LE lineage.

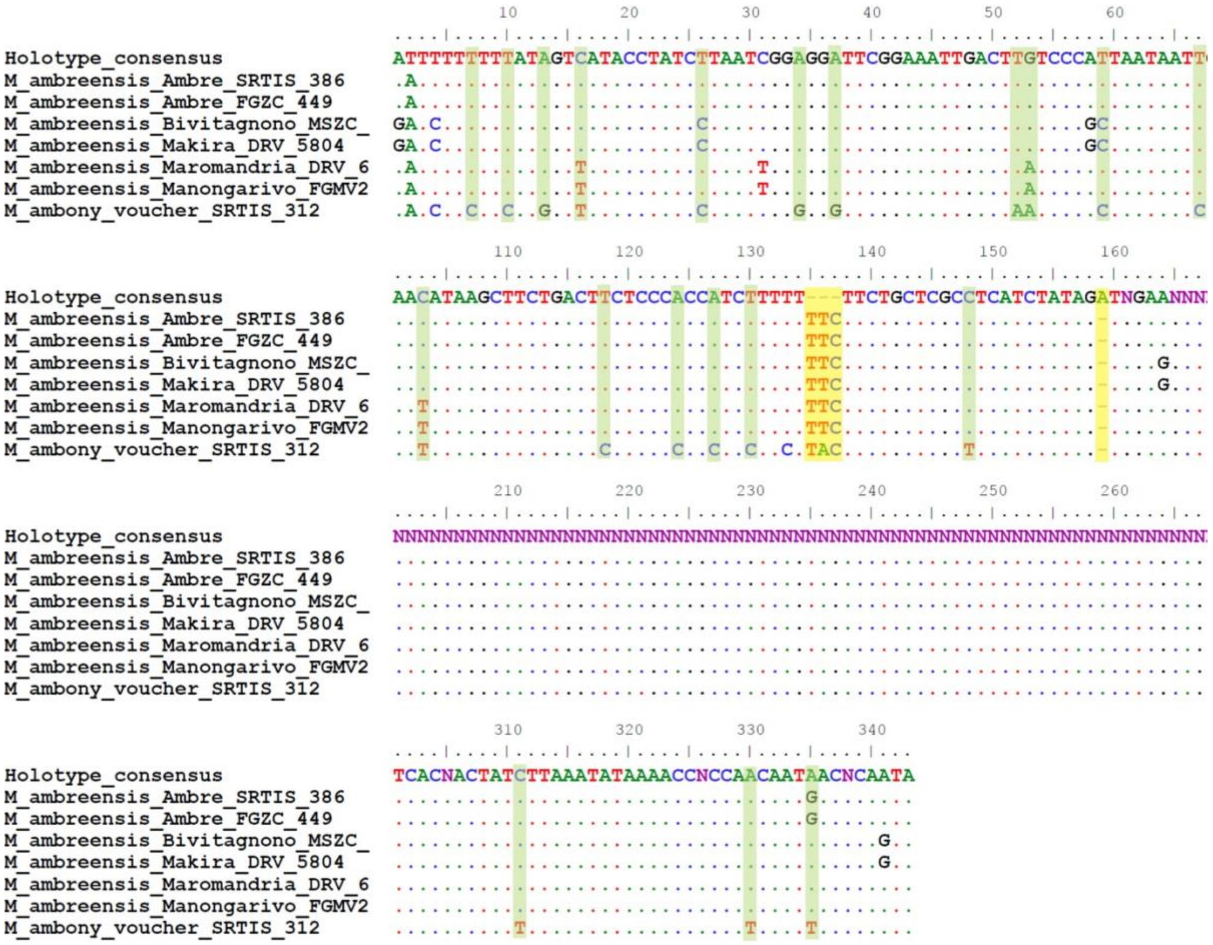

Supplementary Figure S2. Alignment of the cox1 gene, showing differences of the reconstructed sequence of the *Mantidactylus ambreensis* holotype (obtained by targeted enrichment sequencing) in comparison with selected sequences of specimens obtained by Sanger sequencing. Green bars highlight 20 diagnostic alignment positions in which the holotype agrees with (at least the topotypical) specimens of the LE lineage but differs from the HE lineage (that is described herein as *M. ambony* sp. nov.). Yellow bars mark probable assembly errors. Note that in the first two positions, the holotype sequence differs from all reference sequences, which probably is due to sequencing errors.
